# The relationship of lung cancer with menstrual and reproductive factors may be influenced by passive smoking, cooking oil fumes, and tea intake: A case–control study in Chinese women

**DOI:** 10.1097/MD.0000000000008816

**Published:** 2017-11-17

**Authors:** Fei He, Jing-xian Xie, Chun-lan Liu, Wei-min Xiong, Qiu-ping Xu, Zhi-qiang Liu, Tao Lin, Ren-dong Xiao, Xu Li, Lin Cai

**Affiliations:** a Department of Epidemiology; b Fujian Provincial Key Laboratory of Environment Factors and Cancer, School of Public Health, Fujian Medical University, Fuzhou; c State Key Laboratory of Molecular Vaccinology and Molecular Diagnostics, Xiamen University, Xiamen, Fujian; d Department of Obstetrics and Gynecology, Xiamen Maternity and Child Health Care Hospital; e Department of Thoracic Surgery, The First Affiliated Hospital of Fujian Medical University; f Fujian Provincial Key Laboratory of Tumor Microbiology, Fujian Medical University, Fuzhou, China.

**Keywords:** female, lung neoplasms, reproductive history, menstruation

## Abstract

The aim of this study was to investigate the association of menstrual and reproductive factors with risk of lung cancer in women. Potential etiological clues related to lung cancer in women are identified to inform preventive strategies.

Case–control study of 477 newly diagnosed women with lung cancer and 479 age-matched (±2 years) controls. Data on menstrual and reproductive factors and history of oral contraceptive use were obtained on personal interviews using a structured questionnaire. Risk factors were analyzed by unconditional logistic regression analysis.

Maternal age ≥25 years at first birth appeared to protect against female lung cancer [odds ratios (ORs): 0.511, 95% confidence interval (CI), 0.376–0.693]. Age at menopause > 50 years and use of contraceptives was associated with an increased risk of lung cancer in women (OR: 1.471, 95% CI, 1.021–2.119 and OR: 1.844, 95% CI: 1.111–3.061, respectively). Age ≥13 years at menarche was associated with a decreased risk of lung adenocarcinoma (OR: 0.563, 95% CI, 0.317–0.997). There was significant heterogeneity in the levels of cooking oil fume (COF) exposure (*P*_heterogeneity_ = .015). Higher levels of exposure to passive smoking, COF, and lack of tea intake were associated with an increased risk of lung cancer.

Menstrual and reproductive factors are considered to play a role in the development of lung cancer in women. Exposure to passive smoking, COF, and lack of tea intake appeared to significantly modify the relationship.

## 1 Introduction

Lung cancer is an important global public health problem, characterized by delayed diagnosis, early metastasis, high mortality, and poor survival. Incidence of lung cancer and the associated mortality rates have increased over the past 30 years. According to the International Cancer Research Center, the global standardized incidence rates of lung cancer were 50.4/100,000 for men and 19.2/100,000 for women in 2012; the global standardized death rates in the same year were 36.8/100,000 for men and 14.3/100,000 for women.^[1]^ Although the incidence of lung cancer among women is relatively low, it is the most common cause of death among women with malignant tumors.

Smoking is recognized as a major risk factor for lung cancer, especially in men. However, given the same smoking exposure, women tend to have a higher susceptibility to lung cancer than men.^[2]^ Further, nonsmoking women were shown to be more likely to develop lung cancer than nonsmoking men.^[3]^ Adenocarcinoma accounts for approximately 85% of all lung cancer subtypes among nonsmoking women.^[4]^

The gender difference in the incidence of lung cancer may be attributable to the physiological characteristics; higher circulating estrogen levels may render women more susceptible to lung cancer. Epidemiological studies of estrogen as a risk factor for lung cancer have focused on menstrual and reproductive history. Findings pertaining to the contribution of menstrual and reproductive history toward risk of lung cancer have been largely inconsistent.^[5–8]^ Studies that have investigated the association between risk of lung cancer and age at first birth, age at menarche, menopause, use of postmenopausal hormone therapy (HT), and oral contraceptives (OCs) have yielded inconsistent results. Moreover, not all studies have confirmed this relationship, including the large Women's Health Initiative clinical trial.^[9]^ In vivo studies have shown that estrogen can promote proliferation and differentiation of cancer cells in the context of small cell and nonsmall cell lung cancers.^[10]^

The aim of this study was to investigate the relationship between menstrual history, reproductive factors, and lung cancer risk in Han female living in southeast China.

## 2 Methods

### 2.1 Study subjects

As part of an ongoing hospital-based case–control study, we enrolled 477 female patients with lung cancer (confirmed by bronchoscopy or histopathology) at 3 hospitals (the First Affiliated Hospital of Fujian Medical University, the Union Hospital of Fujian Medical University, and Fuzhou General Hospital) between January 2006 and July 2015. The control group consisted of 479 age-matched (±2 years) cancer-free individuals recruited from medical examination centers or hospital nononcology departments during the same period. All subjects were Chinese Han women, who were living in Fujian for > 10 years and were able to answer questions clearly. The study was approved by the Fujian Medical University Ethics Committee. Written informed consent was obtained from all subjects before their enrolment in the study.

### 2.2 Survey methods and variables matching

All epidemiological data were obtained by in-person interviews using a standardized questionnaire. Data on following variables were captured by the questionnaire: smoking status; passive smoking; tea consumption; alcohol intake; exposure to cooking oil fume (COF); history of lung disease; family history of cancer; menstrual history (including age at menarche and age at menopause); birth history (including number of live births, age at first birth, and breastfeeding); contraceptive history (contraceptive usage and hormone usage); and history of gynecological surgery. Categorical variables considered as potential risk factors were stratified into 2 levels (yes or no). Continuous variables considered as potential risk factors were stratified into 2 levels by median values of control group.

Smoking status was defined as individuals who had smoked at least 100 cigarettes during their lifetime. Passive smoking was defined as exposure to other environmental sources of tobacco smoke at home and/or at work for more than 15 minutes per day. To evaluate COF exposure, subjects were asked about the fumes in the kitchen during cooking with a binary response (“no” or “yes”). Tea drinkers were defined as those who consumed at least 1 cup of tea per day, for at least 6 consecutive months. We calculated the body mass index (BMI) as body weight (kg)/height^2^ (m). All data were entered and cleaned using EpiData 3.1 software (Odense, Denmark).

### 2.3 Statistical analysis

SPSS (Version 23.0; IBM SPSS, Inc., Chicago, IL) software was used for data analysis. Distribution of demographic variables in cases and controls was analyzed using Student *t* test and χ^2^ test. The association between exposure to menstrual history, reproductive factors, and risk of lung cancer was evaluated on unconditional logistic regression analysis. Initially, the risk factors for lung cancer were identified by calculating the odds ratios (ORs) and 95% confidence intervals (95% CIs). Ordinal logistic regression analysis was carried out for the association between exposure factors and different lung cancer subtypes. Multivariate logistic regression analysis was carried out to identify the adjusted ORs. All the selected variables were entered as independent variables in the analyses. All tests were 2-sided and statistical significance was defined at *P* < .05.

## 3 Results

### 3.1 The relationship between selected factors and lung cancer

Mean age [± Standard deviation (SD)] of the cases and controls was 56.44 (±10.83) and 56.51 (±10.72) years, respectively. No significant between-group difference in age was observed on Student *t* test (*t* = 0.110, *P* = .913). Mean BMI (±SD) of the cases and controls was 22.66 (±3.52) and 23.09 (±3.19), respectively; the between-group difference was statistically significant (*t* = 2.006, *P* = .045). The pathological subtypes of lung cancer were adenocarcinoma (N = 344), squamous cell carcinoma (N = 40), small cell carcinoma (N = 16), and others (N = 68). There was no statistically significant difference in the distribution of age and marital status between cases and controls (*P* > .05), but there was significant difference in the distribution of BMI, occupation, and educational level (*P* < .05) (Table 1). As expected, passive smoking and COF was associated with an increased risk of lung cancer in women (OR for passive smoking: 2.164, 95% CI: 1.671–2.803; OR for COF: 2.625, 95% CI: 1.908–3.612). Tea consumption was associated with a decreased risk of lung cancer in women (OR: 0.379, 95% CI: 0.282–0.510). Therefore, passive smoking, COF, and tea consumption were further studied in subsequent analyses.

**Table 1:**
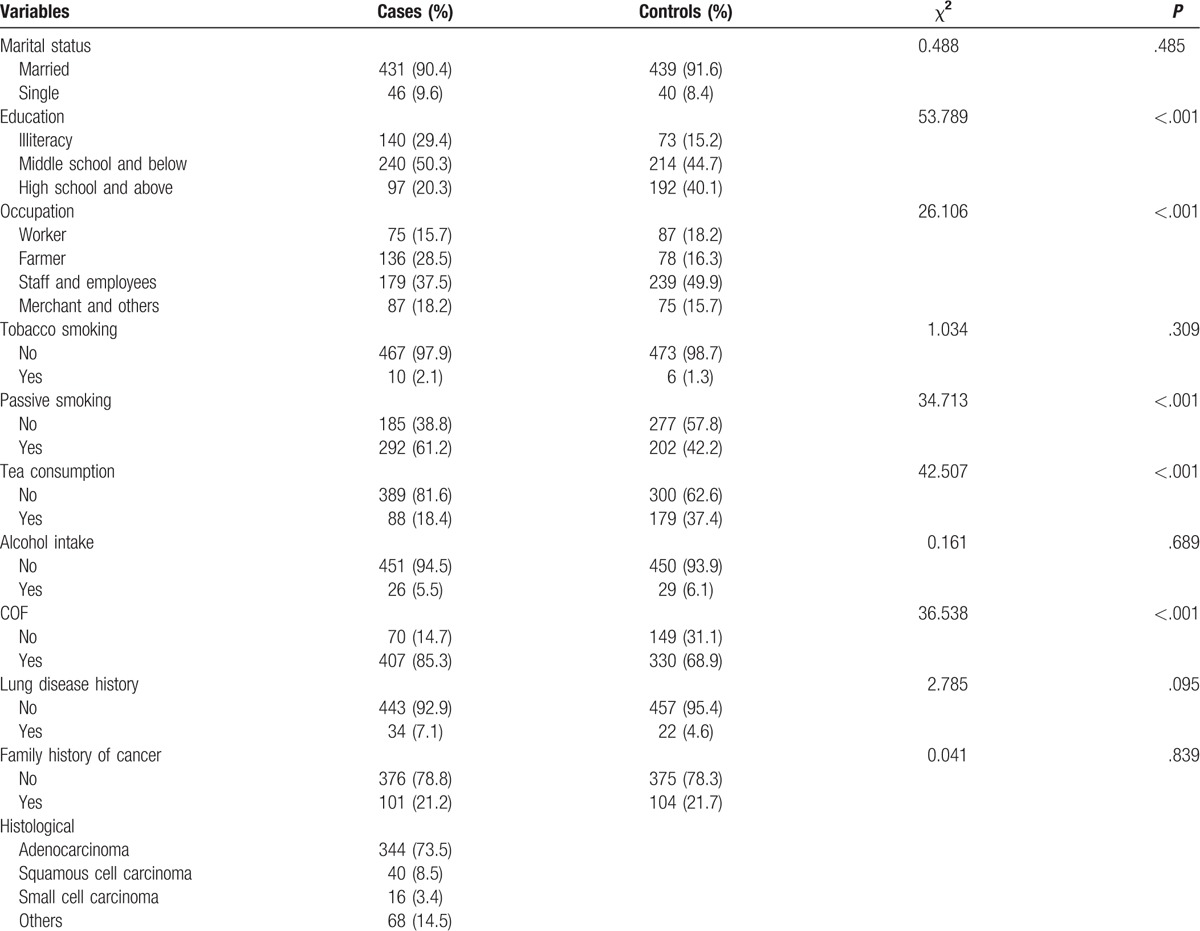
Distribution of selected variables and characteristics in patients with lung cancer and controls.

### 3.2 Association of menstruation history with the histological subtype

After adjusting for age, BMI, education, occupation, marital status, tobacco smoking, passive smoking, alcohol drinking, COF, tea consumption, history of lung disease, and family history of cancer, age at first birth older than 25 years was associated with a decreased risk of lung cancer in women (OR: 0.511, 95% CI: 0.376–0.693); age at menopause older than 50 years and contraceptive use was associated with an increased risk of lung cancer (OR: 1.471, 95% CI: 1.021–2.119) and 1.844 (95% CI: 1.111–3.061), respectively. Age at menarche ≥13 years was associated with a 44% reduction in risk of lung adenocarcinoma and age at first birth ≥25 years was associated with 41% reduction in risk of lung adenocarcinoma; contraceptive use was a risk factor for lung adenocarcinoma (OR: 1.739, 95% CI: 1.006–3.006). Age at first birth ≥25 years was a protective factor for squamous cell carcinoma (OR: 0.260, 95% CI: 0.107–0.631) and for other types of lung cancer (OR: 0.346, 95% CI: 0.184–0.649). For the stratified analysis of squamous cell carcinoma, although some strata lacked enough sample size, we still found that simple tubal ligation was a risk factor (OR: 2.205, 95% CI: 1.071–4.540). Number of live births > 3 (OR: 2.165, 95% CI: 1.170–4.009) and age at menopause >50 years (OR: 2.186, 95% CI: 1.078–4.434) was found to increase the risk of other types of lung cancer. (Table 2). Therefore, age at first birth, age at menopause, and contraceptive usage maybe associated with female lung cancer.

**Table 2:**
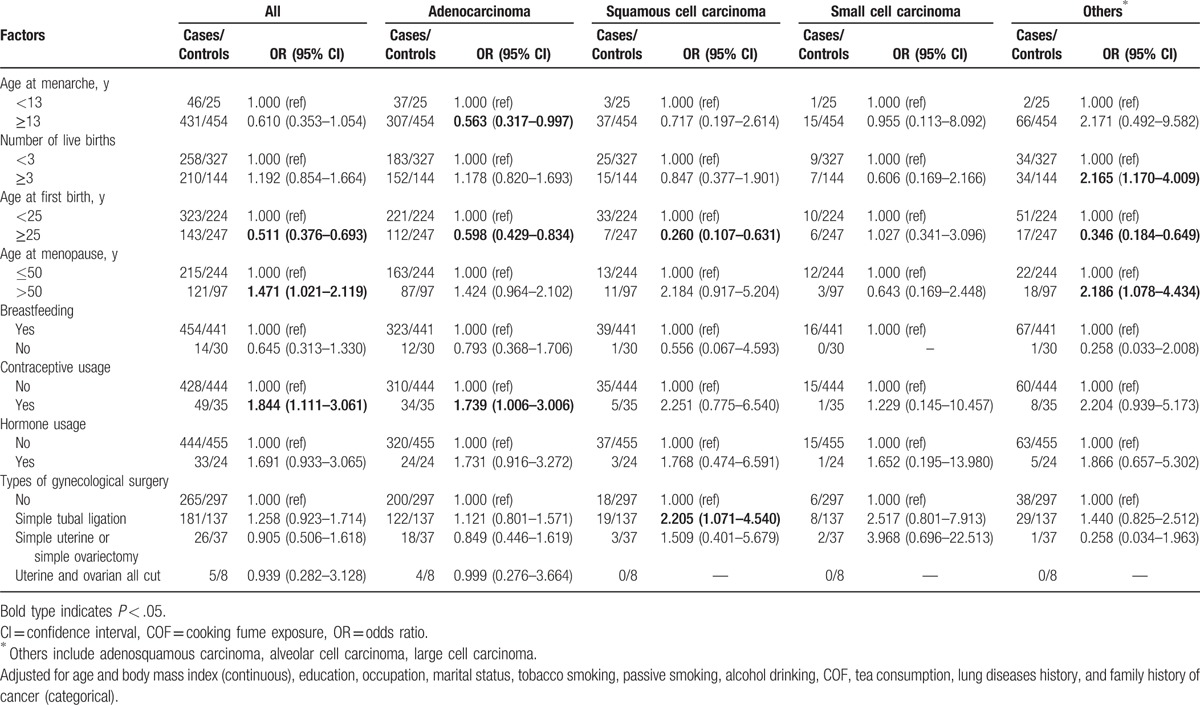
The relationship between menstruation history and the histological subtype of lung cancer.

### 3.3 Effect of passive smoking, COF, and tea consumption

On the basis of these results, we analyzed the effect of menstruation history after stratifying the data based on exposure to passive smoking, COF, and tea consumption. The association between different menstruation-related factors and female lung cancer stratified by passive smoking, COF, and tea consumption is summarized in Table 3. Among nonpassive smokers and non-tea drinkers, age at menarche ≥13 years was a protective factor (ORs: 0.412 and 0.503, respectively, *P* < .05). Irrespective of exposure to passive smoking, COF, and tea, age at first birth seemed to decrease the risk of lung cancer. Among the nonpassive smokers, exposure to COF and non-tea drinkers, age at menopause >50 years was associated with a higher risk of lung cancer (ORs: 1.811, 1.569, and 1.872, *P* < .05). Similarly, in nonpassive smokers and those not exposed to COF, use of contraceptive appeared to increase the risk of lung cancer (OR: 2.332, 95% CI: 1.144–4.757; OR: 3.682, 95% CI: 1.076–12.592). For passive smokers and those not exposed to COF, simple tubal ligation was associated with an increased risk of lung cancer (ORs: 1.615 and 2.453, respectively; *P* < .05). Furthermore, irrespective of the COF exposure, an inverse association between age at first birth and lung cancer was observed; however, the effects between 2 levels were different (*P*_heterogeneity_ = .015).

**Table 3:**
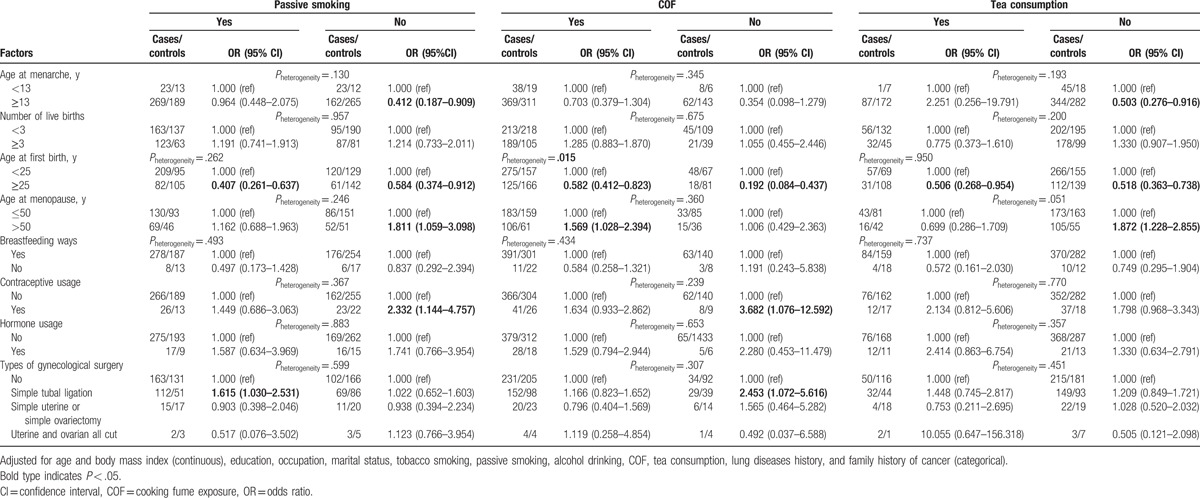
The stratification analyses of passive smoking, COF, and tea consumption on lung cancer.

### 3.4 Combined effects analyses of menstruation factors, passive smoking, COF, and tea consumption

We chose age at menarche, age at first birth, age at menopause, and history of contraceptive use for assessing the combined effect of tea consumption, passive smoking, and COF. Due to inadequate sample size in the various subgroups, the association of age at first birth, age at menopause, history of contraceptive use in combination with tea consumption, and exposure to passive smoking and COF could not be performed. Compared with individuals whose age at menarche was ≥13 years with tea drinking and no passive smoking and no COF exposure, we observed that the ones with menarche at < 13 years of age and only exposed to 1, 2, and 3 factors (1 exposure means passive smoking, COF, or lack of tea intake; 2 exposure means 2 factors among these 3; 3 exposure means exposed to passive smoking and COF and lack of tea intake) (OR: 49.081, 55.971, and 94.921, respectively; *P* < .05) may significantly increase the risk of lung cancer in Fujian Han women. Among women who experienced menarche after the age of 13 years, those who drank tea but were not exposed to passive smoking and COF were at a lower risk of lung cancer than those exposed to passive smoking but not to COF and those exposed to both passive smoking and COF (ORs: 19.189, 32.236, and 80.043, respectively; *P* < .05).

## 4 Discussion

In the current study, we selected the variables age at menarche, number of live births, age at menopause, breastfeeding, contraceptive usage, hormone usage, and types of gynecological surgery to investigate the effects of estrogen-related factors effects on female lung cancer. Our results indicated that women who bear their first child after the age of 25 years may have a lower risk of lung cancer (OR: 0.511, 95% CI: 0.376–0.693); those who experience menopause after the age of 50 years and those who have a history of contraceptive use were at a higher risk of lung cancer (OR: 1.471, 95% CI, 1.021–2.119; OR: 1.844, 95% CI: 1.111–3.061). Those who experience menarche after 13 years of age were at a lower risk of lung adenocarcinoma (OR: 0.563, 95% CI: 0.317–0.997). The heterogeneity in exposure to COF (*P*_heterogeneity_ = .015) and the fact that higher levels of exposure to passive smoking, COF, and lack of tea intake increased the risk of lung cancer indicated that passive smoking, COF, and abstinence from tea would significantly modify the association of menstrual and reproductive factors with lung cancer among Chinese women.

Brinton et al^[11]^ studied a cohort of 1,850,179 NIH-AARP (National Institutes Of Health-American Association Of Retired Persons) diet and health study, and found that after adjusting for variables such as smoking, passive smoking, and cooking fumes, late menarche protected against lung adenocarcinoma in women (OR: 0.72, 95% CI: 0.56–0.93, *P*_*trend*_ < .01). The findings are consistent with our results. However, some studies^[9,12]^ appeared to refute the association of age at menarche and risk of lung cancer. We presume an increased secretion of estrogen at menarche and during ovarian and endometrial cyclical changes. In vitro and animal models have found estrogens to promote lung tumor growth; further, estrogen receptor antagonists were shown to significantly inhibit the growth of nonsmall cell lung cancer cell lines.^[13]^ A later age at menarche in effect implies postponement of the estrogen surge that may serve to decrease the risk of lung cancer.

In the current study, we divided into 2 groups to assess the relationship between age at menopause and lung cancer risk. We found that age < 50 years at menopause may decrease the risk. Similar results have been reported elsewhere. This implies that late menopause increases susceptibility to lung cancer.^[14–16]^ In recent studies, menopausal women aged >60 years with lung cancer were shown to have a higher expression of aromatase (up to 84.76%), which is a key enzyme for synthesis of estrogen.^[17]^ Especially, serum concentration of estrogen in patients with lung cancer was significantly higher than those in healthy controls.^[18]^ Female menopause entails a decline in ovarian function, a change that gradually develops over the last few periods. Postmenopausal women have significantly reduced estrogen levels. The above findings suggest that prolonged in vivo exposure to high estrogen levels increases susceptibility to lung cancer.

We also observed a protective effect of late age at first birth against lung cancer. This phenomenon is also observed with respect to chronic lung disease, diabetes, and high blood pressure.^[19]^ Age at first live birth was not associated with lung cancer risk in most studies.^[5,8,16,20–23]^ A few^[24,25]^ reported an increased risk with older age at first live birth, while others ^[12,15,24]^ have reported an inverse association. With regard to OC use, this study showed a positive association of contraceptive usage with lung cancer risk. This is consistent with findings from the Nurses’ Health Study^[25]^ in which duration of OC use > 5 years was associated with a slightly increased risk of lung cancer. However, most observational studies have found no association with lung cancer risk,^[21,22,26]^ while Kreuzer et al^[12]^ observed a reduction in lung cancer risk.

Findings of the association between menstrual history and lung cancer risk have been inconsistent; the possible reasons may be differences in the study population, including the smoking rates and ethnic differences. Smoking is a major risk factor for lung cancer, and smoking levels in the study population will have a mixed effect on the results. There may also be racial differences in the study populations; The sample size of this study has a greater impact; and concomitant use of different variables in the subgroup analyses is also liable to affect the results.

The underlying mechanism of the association between menstrual and reproductive history and lung cancer is not clear. Estrogen may play an important role in the body's normal physiology as well as in tumorigenesis. It promotes the development of the female reproductive system, maintenance of secondary sexual characteristics and calcium metabolism, and can lead to human breast cancer,^[27]^ endometrial cancer,^[28]^ and other cancers. Lungs are usually not the target organ of female hormones, but estrogen and progesterone receptors are detectable in normal lung and cancerous tissues.^[29]^ Most of the previous studies have focused on the relationship between menstrual history and lung cancer. In this study, we further analyzed the combined effects of environmental risk factors and menstrual history on lung cancer. Our results suggest passive smoking, COF, and tea drinking as potential modifiers of the association of menstrual and reproductive factors with lung cancer in Chinese women.

This study also has some limitations. First, as a case–control study, recall bias possibly exists. To reduce the impact of recall bias on the results, we chose all newly diagnosed cases. Moreover, all interview procedures were standardized, the same interviewers were used at all study sites, and the information was recorded on the basis of objective indicators during the investigation. Second, selection bias would be another major concern for this study. However, all participants were recruited by the same criteria in the different hospitals, which could reduce the possibility of selection bias. Due to the limited number of cases, the association between some of the variables (such as use of OCs and hormone replacement therapy) and lung cancer could not be explored. Therefore, large sample studies are expected in the future.

## 5 Conclusion

Menstrual and reproductive history may be associated with the development of lung cancer in women. In particular, factors such as age at menarche, age at menopause, age at first gestation, and contraceptive usage may play an important role in the development of lung cancer. Research into the relationship between female hormones and lung cancer has evoked much interest; however, the results of published studies are not consistent and the underlying mechanism is still unclear. Well-designed prospective studies with use of molecular biology markers and larger sample size will help unravel the relationship between female hormones and lung cancer.

## Acknowledgments

We thank all staff members from the Department of Thoracic Surgery, The First Affiliated Hospital of Fujian Medical University. We would also like to express our appreciation to the patients who participated in the study.
